# Considering the cumulative risk of mixtures of chemicals – A challenge for policy makers

**DOI:** 10.1186/1476-069X-11-S1-S18

**Published:** 2012-06-28

**Authors:** Denis A Sarigiannis, Ute Hansen

**Affiliations:** 1 European Commission – Joint Research Centre, Institute for Health and Consumer Protection, Chemical Assessment and Testing, via E. Fermi 1, 21027 (VA), Italy; 2 Aristotle University of Thessaloniki, Chemical Engineering Department, Environmental Engineering Laboratory, University Campus, Bldg. D, 50441 Thessaloniki, Greece

## Abstract

**Background:**

The current paradigm for the assessment of the health risk of chemical substances focuses primarily on the effects of individual substances for determining the doses of toxicological concern in order to inform appropriately the regulatory process. These policy instruments place varying requirements on health and safety data of chemicals in the environment. REACH focuses on safety of individual substances; yet all the other facets of public health policy that relate to chemical stressors put emphasis on the effects of combined exposure to mixtures of chemical and physical agents. This emphasis brings about methodological problems linked to the complexity of the respective exposure pathways; the effect (more complex than simple additivity) of mixtures (the so-called 'cocktail effect'); dose extrapolation, i.e. the extrapolation of the validity of dose-response data to dose ranges that extend beyond the levels used for the derivation of the original dose-response relationship; the integrated use of toxicity data across species (including human clinical, epidemiological and biomonitoring data); and variation in inter-individual susceptibility associated with both genetic and environmental factors.

**Methods:**

In this paper we give an overview of the main methodologies available today to estimate the human health risk of environmental chemical mixtures, ranging from dose addition to independent action, and from ignoring interactions among the mixture constituents to modelling their biological fate taking into account the biochemical interactions affecting both internal exposure and the toxic potency of the mixture.

**Results:**

We discuss their applicability, possible options available to policy makers and the difficulties and potential pitfalls in implementing these methodologies in the frame of the currently existing policy framework in the European Union. Finally, we suggest a pragmatic solution for policy/regulatory action that would facilitate the evaluation of the health effects of chemical mixtures in the environment and consumer products.

**Conclusions:**

One universally applicable methodology does not yet exist. Therefore, a pragmatic, tiered approach to regulatory risk assessment of chemical mixtures is suggested, encompassing (a) the use of dose addition to calculate a hazard index that takes into account interactions among mixture components; and (b) the use of the connectivity approach in data-rich situations to integrate mechanistic knowledge at different scales of biological organization.

## Background

The current paradigm for the assessment of the health risk of chemical substances focuses primarily on the effects of individual substances for determining the doses of toxicological concern in order to inform appropriately the regulatory process. Given the recently increased public awareness on the link between environmental conditions and public health, the policy-making and environmental management processes have an enhanced need for health and safety data. In Europe, this was signalled in the 6th Environmental Action Plan (2001-2010), where for the first time the issue of environment and health was identified as a key determinant of sustainability. Since then, a number of legislative initiatives have been undertaken in the European Union with a scope to reducing the potential adverse effect of environmental pressure on public health as a key dimension for ensuring sustainability. These include

(a) Consumer Policy and REACH, the current chemical safety regulation in the European Union, requiring sound data on chemical safety of consumer products [1];

(b) Environment & Health Action Plan putting particular emphasis on mixture effects in various environmental matrices [2];

(c) Food safety policies regarding chemicals in food and food contact materials [3].

These policy instruments place varying requirements on health and safety data of chemicals in the environment. REACH focuses on safety of individual substances; yet all the other facets of public health policy that relate to chemical stressors put emphasis on the effects of combined exposure to mixtures of chemical and physical agents. This emphasis brings about methodological problems linked to the complexity of the respective exposure pathways; the effect (more complex than simple additivity) of mixtures (the so-called 'cocktail effect'); dose extrapolation, i.e. the extrapolation of the validity of dose-response data to dose ranges that extend beyond the levels used for the derivation of the original dose-response relationship; and the integrated use of toxicity data across species (including human clinical, epidemiological and biomonitoring data).

In this paper we give an overview of the main methodologies available today to estimate the human health risk of environmental chemical mixtures, ranging from dose addition to independent action, and from ignoring interactions among the mixture constituents to modelling their biological fate taking into account the biochemical interactions affecting both internal exposure and the toxic potency of the mixture. We discuss the possible options available to policy makers and the difficulties and potential pitfalls in implementing these methodologies in the frame of the currently existing policy framework in the European Union. Finally, we suggest a plausible scenario for policy/regulatory action that would facilitate the evaluation of the health effects of chemical mixtures in the environment and consumer products.

## Mixture toxicology methods

**Figure 1:**
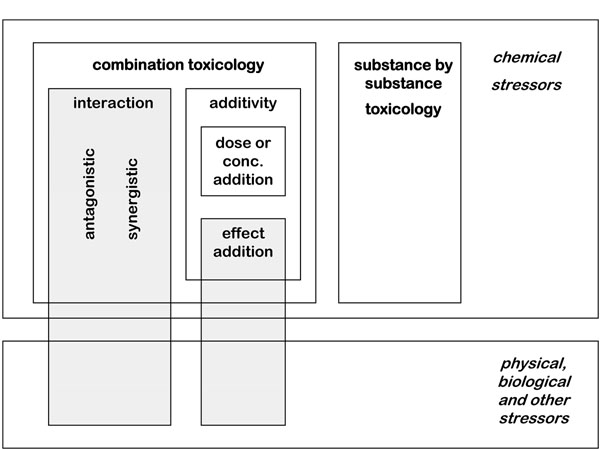
**Scheme of risk assessment approaches** Scheme of risk assessment approaches. Currently methodology is available for substance by substance toxicology and the assessment of mixtures of substances with the same mechanism of action for which dose or concentration addition is applied.

As described in the text above, from a mixture toxicology point of view each methodology to predict cumulative effects or to detect interactions has limited applicability under specific conditions that cannot be generalized. Information on mode and mechanism of action for each active substance in the mixture is needed to decide whether to use dose or effect addition, while biochemical and molecular pathway information regarding enzyme activity and the ADME (absorption – distribution – metabolism – excretion) properties of the substances are needed to determine the existence or not of these processes and the level of biochemical interactions among them. In risk assessment and management, however, a more pragmatic viewpoint is taken, and given the overall uncertainties of the process an approximation to the cumulative effects is usually good enough, for example a worst-case, but not overly conservative, estimate. One key issue is, nonetheless, the need to render the risk assessment transparent by recognizing and reporting explicitly on the uncertainty and the assumptions associated with each of the risk assessment methodologies described below and in the next chapter (see Figure 1).

### Hazard index

The hazard index (HI) of a mixture of chemicals is the sum of the compound-specific hazard quotients (HQ_i_), which are calculated as the ratio of the exposure (e.g. the daily intake of a substance) to the dose of no concern (i.e. the exposure above which adverse effects on human health can be expected) [4-7]. This reference dose (ref_i_) can be, for example, the acceptable daily intake (ADI) or the acute reference dose (ARfD).

The hazard index for a mixture of several substances S_i_ (i = 1,2,…n) can be calculated as(9)

The hazard index method does not allow prediction of the overall health effect of the combined substances but adds the strength of risk (roughly estimated), attributable to each component of the mixture. If the index exceeds unity the concern is the same as if an individual chemical exposure exceeded its acceptable level by the same proportion [4]. The hazard index can be used to identify the most risky substances in a mixture, i.e. the chemicals that have the highest health risks based on toxic potential and estimated or measured exposure. Feron et al [5] propose to use the Mumtaz-Durkin and Mumtaz et al [6,7] weight of evidence approach, which is based on the hazard index concept but additionally considers evidence for interactions for the selection of the most risky chemicals or groups of chemicals in a complex mixture (see below).

Risk prediction using the HI approach is based on a comparison of specific exposure to the substance-specific exposure limit, ref_i_, for each component of the mixture. If this exposure limit is derived from testing effects on an identical physiological target, e.g. effects on a certain enzyme measured under defined conditions, the result obtained is equivalent to the outcome of the dose summation method. A kind of effect addition is performed through the HI calculation if the ref_i_ values refer to a more general endpoint, induced by similar or dissimilar mechanisms, e.g. if ref_i_ values are threshold concentrations above which adverse effects on the liver are expected. It allows prediction of even more general risk indices. If for the first compound of a mixture a ref_i_ is available for lung cancer, for the second the ref_i_ refers to liver cancer and the third component is known to enhance the risk to a third type of cancer, a risk index, named Health Risk Index, can be calculated for the general category “cancer”. It should be noted that exp_i_ and ref_i_ should refer to the same time scale of exposure in terms of chronic versus acute exposure and toxicity, and to the same uptake route, because, for example, the amount of a toxic substance taken up with food may not be directly compared to the toxicity threshold derived from an inhalation study.

The hazard index concept has the advantage that it is transparent and easy to apply [8-10]. The disadvantage is that the reference to which exposure is related does not solely reflect the toxicity of the substance but is derived by using uncertainty factors which are not fully data based but may incorporate significant policy-driven assumptions: For calculating reference concentrations of no concern (ref_i_) the NOAELs usually are divided by a factor accounting for the uncertainty of the generalisation of the NOAEL as specified also in the respective technical annex of the REACH Regulation. If the NOAEL is known only for a low number of species/trophic groups this assessment factor is high and the resulting threshold is low. If testing has been performed for several species and trophic groups extrapolation from test species to humans is more reliable. In this case the assessment factor is lower so that the reference value deviates to a lesser extent from the NOAEL.

### Point of departure index

The point of departure index (PODI) method does not have the disadvantage of the hazard index, as exposure is compared to a concentration level reflected by toxicity data. The point of departure index is the sum of the exposure of each compound divided by its respective point of departure (POD),(10)

where the POD can be a NOAEL, the dose at which a toxic effect becomes biologically significant, or a benchmark dose [11,12]. The benchmark dose method is a statistical tool fitting a mathematical model to all the dose-response data within a study and thus more biological information is used for the derivation of the POD compared to the traditional NOAEL derivation [12]. The benchmark dose (BMD) method provides additional information regarding its uncertainty but high quality dose response curves are required in order to provide estimated BMDs with small confidence intervals [10]. For the evaluation of potential risk the PODI of a mixture is compared to an agreed group safety factor. This factor is often 100 and the product of PODI and the uncertainty factor should be <1 [10].

### Margin of exposure

The margin of exposure (MOE) of a substance is the NOAEL divided by exposure so that the combined margin of exposure of a mixture (MOE_mix_) can be calculated as(11)

The margin of exposure index of a mixture is compared to an agreed acceptable threshold. According to EFSA [10] there are no established criteria for the magnitude of an acceptable MOE_mix_ for mixtures of chemicals but it is widely accepted that at a MOE_mix_ higher than the uncertainty factor of 100 the conclusion can be drawn that the risk of toxicity is unlikely.

### Cumulative risk index

The cumulative risk index (CRI) combines MOEs for chemicals with different uncertainty factors. The risk index (RI) of a single chemical is the reciprocal of the hazard quotient and is calculated as(12)

where UF is the uncertainty factor mentioned above (e.g. [10]). The reference dose (ref_i_) can be, for example, the acceptable daily intake, ADI. The cumulative risk index (CRI) of a mixture of chemicals is defined as(13)

The cumulative risk index is the reciprocal of the hazard index (equation 9).

### Toxic equivalency factors

The Toxic Equivalence Factor (TEF) is a specific type of relative potency factor (see section 2.2) formed through a scientific consensus procedure [10]. Based on the assumptions of a similar mechanism of action of structurally related chemicals and parallel concentration (or dose) response curves, TEFs were first developed for polychlorinated dibenzo-*p*-dioxins (PCDDs) and polychlorinated dibenzofurans (PCDFs) and polyaromatic hydrocarbons (PAHs). The total toxicity of the mixture is assessed in terms of the toxicity of an equivalent concentration of an index compound. The total equivalent quantity *TEQ* is estimated by summation of the concentrations (or doses) of mixture components *c_i_* multiplied by the respective TEF*_i_*:(14)

The TEF/RFP approach is equivalent to the MOE approach described above (see Section 3.3). The major difference is the stage at which to extrapolate from experimental data to humans: for each chemical separately (allowing different assessment factors for each chemical) in the case of MOE; or after combining their doses/effects (using thus one assessment factor for all chemicals in the mixture) in the case of the TEF/RFP approach.

## Methods for risk assessment of mixtures taking into account interactions

Toxicant interactions may take place during any of the processes that affect the toxic potency of a single compound: adsorption, distribution, metabolism, excretion and activity at the receptor site(s). They may interact chemically, and they may interact by causing different effects at different receptor sites [4]. Interactions can be assumed to occur frequently and often are dose-dependent but, according to EFSA [7], there is no standard study design to evaluate the potential interaction of compounds. Evidence exists that mixtures having additive toxicity at low, environmentally relevant concentrations show synergism at higher exposure. This was shown for effects of pesticides mixtures on a target enzyme extracted from tissue of salmon [13]. At EC50 synergy effects were higher compared to 0.4 and 0.1% of EC50, respectively.

Most commonly, dose additivity is considered the expected outcome when testing for the effects of chemical mixtures; any deviations thereof are deemed interactions. However, this is not necessarily the case, as deviations from additivity may also be observed in absence of interactions [14]. Experimental observations of deviations from additivity have been reported, although Kortenkamp et al. [15] report them as being quite rare. Nesnow et al. [16] analysed mixture effects of five polycyclic aromatic hydrocarbons on lung tumours in A/J mice, with mixture ratios representative of ambient air levels of these carcinogens. At low doses greater than additive effects were seen, whereas at high doses the observed responses fell short of additivity expectations which were derived from independent action in an effect surface analysis. However, the observed deviations from effect addition were rather small.

Another example is the study by Walker et al. [17] who employed two year rodent cancer bioassays with female Harlan Sprague-Dawley rats given 2,3,7,8-tetrachlorodibenzo-*p*-dioxin (TCDD), 3,3',4,4',5-pentachlorobiphenyl (PCB-126), 2,3,4,7,8-pentachlorodibenzofuran (PeCDF), or a mixture of the three compounds. The three chemicals, both singly and in combination, induced hepatic, lung and oral mucosal neoplasms. A re-analysis of the data, without utilizing the WHO TEF values [10] but by employing the concept of dose addition directly, showed that the experimentally observed tumor incidences fell short of those anticipated by dose addition [15].

There are a few examples from the area of endocrine disruption that indicated antagonisms in the joint effects of estrogenic agents [18], but these deviations were rather small. Similarly, the study by Hass et al. [19] on the feminizing effects of androgen receptor antagonists on male offspring of dams dosed during gestation indicated a weak synergism with respect to induction of nipple retention. Similar deviations from additivity were not observed with other endpoints evaluated in the same study.

Even though on many an occasion deviation from dose additivity may not be easy to observe at low, environmentally relevant doses, this may be mostly due to the sensitivity and the binary (effect or no effect) nature of classic toxicological tests. The advent of –omics and in particular transcriptomics opens the way to identifying deviations from the assumption of additive response to co-exposure to chemical substances. Toxicogenomics seems to be the appropriate screening method for assessing biological effects of complex chemical mixtures, allowing us to review the whole spectrum of potential biological response rather than focusing on a predefined number of endpoints as in classical toxicological analysis. Applying such techniques Coccini et al. [20] showed a distinct modulation in the muscarinic receptor density and gene expression associated with neurological response in rats co-exposed to methyl mercury and PCB153 perinatally. Cimino Reale et al. [21] studied comparatively the mixture effects of typical mixtures found in indoor air in Europe and of a mixture of polyaromatic hydrocarbons (PAHs) sampled in Milan, Italy. Large sets of genes modulated by exposure to different air mixtures were profiled and common biochemical pathways and specific molecular responses were identified. Indoor air mixtures induced a higher gene modulation than PAHs, confirming major differences in the toxic mode of action of the two mixtures. Indoor air induced primarily modulation of genes associated with protein targeting and localization including in particular cytoskeletal organization; PAHs modulated mostly the expression of genes related to cell motility and gene networks regulating cell-cell signaling, as well as cell proliferation and differentiation. These results provide biological information useful for articulating mechanistic hypotheses of exposure to xenobiotic mixtures and physiological responses.

One way to deal with the general lack of knowledge about interactions in a cumulative risk assessment context is to use an additional uncertainty factor accounting for potential synergy effects. As mentioned above, NOAELs usually are divided by assessment factors considering, for example, the uncertainty of extrapolation of data gained with certain test organisms in the lab to toxicity thresholds applicable for human health protection (factor = 10) and the between humans differences in sensitivity (factor = 10). Increasing the uncertainty factor by a factor of 10 and thus accounting for interactions of chemicals in a mixture would cover a tenfold increase in mixture toxicity due to interactions between the mixture components. Not much is known about the significance and extent of synergy effects and it is unclear whether an uncertainty factor of 10 would be protective or over-protective. Currently no specific assessment factor for mixtures is employed in the traditional chemical-by-chemical risk assessment [5].

### Interactions based hazard index

A modified hazard index approach has been proposed [10], using the hazard index developed for additive effects as a basis and accounting for interactions by multiplying the HI with a factor reflecting both the uncertainty and the strength of evidence that interactions take place. The interaction-based hazard index (HI_I_) is calculated as:(15)

where UF_I_ is the uncertainty factor for interactions. WOE_N_ is a numerical weight-of-evidence score reflecting the strength and consistency of evidence for interactions scaled to reflect the relative importance of the component exposure levels. The method is based on the procedure proposed by Mumtaz and Durkin [6]. Further developments of the HI approach yielded the following equation for HI_INT_, the hazard index modified by binary interactions data [10], applying the weight-of-evidence procedure to modify each HQ instead of modifying the sum of the HQ values of the mixture:(16)

where HI_INT_ is the HI modified by binary interactions data, HQ_i_ is the hazard quotient for chemical *i*, f_ij_ is the toxic hazard of the *j^th^* chemical relative to the total hazard from all chemicals potentially interacting with chemical *i* (thus *j* cannot equal *i*), M_ij_ is the interaction magnitude, the influence of chemical *j* on the toxicity of chemical *i*, B_ij_ is the score for the strength of evidence that chemical *j* will influence the toxicity of chemical *i*, and θ_ij_ represents the degree to which chemicals *i* and *j* are present in equitoxic amounts. How to derive B_ij_, f_ij_, M_ij_ and θ_ij_ is described in detail in EPA [10].

### Excluding interactions – the isobole method

Since both the dose addition and the effect summation methods are applicable only under the precondition that interactions are absent, methods suitable for examining whether all mixture components act without diminishing or enhancing the effects of each other play an important role in current state of the art mixture toxicology. The isobole method [10] is the most common criterion for judging whether interactions between similarly acting chemicals have taken place in a mixture experiment [13]. Bosgra et al. [13] give an overview of the method and discuss its applicability. The isobole method is based on the following approach:

In the case of zero-interaction(17)

where d_1_ and d_2_ are doses of the substances S_1_ and S_2_, respectively, present in a particular combination, and d_1_’ and d_2_’ represent the doses that, if applied individually, result in the same magnitude of effect as the combination. If the mixture has the same effect but the effect is reached with lower d_1_/d_1_’ and/or d_2_/d_2_’ ratio(s) the left part of equation 17 is smaller than 1, reflecting synergy as an enhancement of toxic potency due to interaction of the mixture components. Any deviation from 1 denotes a deviation from additivity [11].

### Modelling interacting mixtures

In the past 15 years or so, physiologically based pharmacokinetic/pharmacodynamic (PBPK/PD) modeling has been applied to the toxicological interactions of chemical mixtures. A comprehensive review on these studies was conducted by Reddy et al. [22]. Progress in the application of PBPK modeling to chemical mixtures has followed different phases from simple binary pharmacokinetic and pharmacodynamic interactions to more and more complex mixtures. First, PBPK modeling of binary chemical mixtures became necessary because of pharmacological or toxicological interactions. Second, as investigators became interested in mechanisms of toxicological interactions, the advances of physiologically based pharmacodynamic (PBPD) modeling formed a natural course of development of this area. Third, when more and more sophistication was incorporated into PBPK modeling, novel approaches towards modeling complex chemical mixtures were developed. Since 1996 when the Food Quality and Protection Act was enacted, the United States Environmental Protection Agency (US EPA) began to give active consideration to cumulative risk assessment taking into account toxicological interactions. Thus, the application of PBPK modeling in cumulative risk assessment became an active area of research [23].

Mumtaz et al. [6] in an earlier review of PBPK modeling of chemical mixtures indicated that the “first example” of PBPK modeling of a “chemical mixture” actually involved one chemical, n-hexane, and its metabolites, methyl n-butyl ketone (MnBK) and 2,5-hexanedione (2,5-HD); thus, it is a kind of “one-chemical mixture.” This particular PBPK model for n-hexane and its metabolites incorporated three inhibitory interactions: (1) hexane and MnBK are competitive substrates for w-1 oxidation; (2) MnBK and 2,5-HD are competitive substrates for oxidation; and (3) 2,5-HD acts as a product feedback inhibitor [24]. The findings of this modeling study were intriguing and they explained some of the most interesting and complex toxicological and pharmacokinetic behaviors of n-hexane in animals [7].

Pioneering efforts in the PBPK modeling of more complex chemical mixtures were from a research group led by Krishnan and various colleagues in Canada; two comprehensive reviews of work up to 1994 are available [25,26]. Earlier work from this group concentrated on interactions and PBPK modeling between two chemicals [27,28] . As progress was made, these investigators began to build up the mixtures and devoted their effort to PBPK modeling of more and more complex chemical mixtures [29-32]. PBPK modeling of a ternary mixture on toluene, m-xylene, and ethylbenzene was studied and reported by Tardif et al. [29], and the interaction mechanism involved was competitive inhibition. Subsequently, Haddad et al. [30] applied this interactive PBPK model to the calculation of the biological hazard index (BHI). BHI, defined as the biological level tolerable to exposure to mixtures, is traditionally calculated in an analogous way to the hazard index under the additivity assumption [30-33]. However, Haddad et al. [30] incorporated toxicological interaction by using PBPK modeling to obtain “simulation concentrations” (SC) and modified the hazard index calculation according to the following equation:(18)

where BHI and SC are as defined before and BEI refers to the concentration or excretion rate of a biomarker in a healthy worker exposed to the occupational threshold limit value (TLV). In doing so, Haddad et al. [30] applied interactive PBPK modeling of a chemical mixture into the risk assessment process. Using the same principle and similar technique, researchers at Colorado State University studied PBPK modeling of two ternary mixtures [trichloroethylene (TCE), tetrachloroethylene (PERC), methyl chloroform (MC) and toluene, ethyl-benzene, and xylenes] to respectively enhance the concept of “interaction thresholds” and modify and improve the “Mixture Formula” risk assessment by using an interactive PBPK modeling approach [34-36].

Haddad et al. [31]also studied the PBPK modeling of a four-chemical mixture involving benzene, toluene, ethylbenzene, and m-xylene. In general, the incorporation of the interaction mechanism, at the level of the liver metabolic enzyme inhibition, is similar to those described above for binary and ternary mixtures, albeit a bit more complicated. In Europe, Sarigiannis and Gotti [37] studied a similar group of volatile organic chemicals including benzene, ethylbenzene, toluene and all the family of xylenes (m-, o-, p-) extending the previous work to tackle the chemical interactions and the biochemical processes influencing the rate of metabolism of the mixture compounds. They brought the methodology one step further by taking into account the morphology of the liver and its relationship with metabolic function, as well as the mechanism of competitive inhibition of metabolism to estimate the biologically effective dose (BED) of the mixture components and their primary metabolites and then related BED for the sum of benzene metabolites (the main carcinogenic compounds in the mixture) to epidemiological and clinical observations of health outcomes (leukemia incidence). This allowed them to derive new dose-response relationships relating the observed health response to the total internal dose of the metabolites at the target tissue (bone marrow in this case), rather than to the external environmental concentrations (doses) of the chemicals in the mixture. The result was a more biologically plausible dose-response curve that captures better the physiological response to toxic insults at low doses and long-term (chronic) exposure.

As PBPK modeling of chemical mixtures progresses towards involving more and more components, a natural course of development is that investigators will attempt to tackle the real-world complex chemical mixtures. Verhaar et al. [38] proposed the incorporation of lumping analyses (a chemical engineering technique used in petroleum engineering processes) and quantitative structure-activity relationships (QSAR) to PBPK modeling. The idea was that each of the three techniques would serve its unique function in the overall goal of predicting some aspects of the chemical mixtures of interest. Thus, QSAR analysis can be used to predict needed physicochemical and toxicological parameters for unknown compounds or for surrogate compounds (from lumping); lumping analysis can drastically reduce the complexity of the description of a mixture; and PBPK/PD modeling can be used to describe the pharmacokinetics, and possibly pharmacodynamics, of an ensemble of compounds or lumped pseudocompounds, including possible interaction effects.

### Grouping of chemicals by health endpoint

There is no established methodology available allowing assessment of the endpoint-specific health risk due to a combination of chemicals having different modes of action and affecting the same endpoint. Although the independent action method for response or effect addition described in detail earlier in this paper allows calculation of summed effects of mixtures of chemicals acting via independent pathways, the applicability of this method for integrated health impact assessment of environmental chemical stressors is limited for the following reasons. First, dose-response relationships for the respective effect are required for each compound of the mixture; these are, in many cases, available for effects on *in vitro* systems or test organisms but lacking for human health endpoints. A further complication results from the specificity of the uptake route of the dose response relationship. A further reason for the limited applicability is that the response addition approach can be used under the precondition that interactions can be excluded. Methods used to test whether no-interaction can be assumed usually require dose-response-algorithms, which are not available for many substance-endpoint combinations.

The hazard index and the interactions-based hazard index method, described above, are applied for assessing potential health risks due to mixtures of chemicals affecting a given health endpoint by acting with the same or differing mode of action. Exposure is compared to a reference exposure assumed to be acceptable in terms of risk to human health. Although the hazard index and related indices mentioned above are not suitable for giving information about the number of cases of impaired health to be expected for a certain complex exposure or policy scenario they represent an important tool for risk assessment and help to decide whether exposure of the population or subgroups to a certain mixture of chemicals should give rise to concern.

For pesticides the suitable substance specific limit value, i.e. the reference level (ref_i_) of no concern, usually is the accepted daily intake rate (ADI) relevant to human health but not to certain health endpoints. Calculating the endpoint specific health risk index for a mixture of pesticides by summing up the exposure to limit value ratios requires health endpoint specific limit values assumed to be protective for the selected endpoint. This kind of reference value, e.g. cancer health risk limit values for humans, is usually not available for pesticides. There are two possibilities to deal with this problem: The first is to identify compounds of the mixture affecting the same health endpoint on the basis of available evaluations, to relate exposure to generic ADIs instead of endpoint specific limit values, and then sum up the ratios to derive the index value. The second possibility is to identify compounds of the mixture showing effects when tested with a certain indicator system (e.g. genotoxicity tests), to relate exposure to the substance specific NOAEL derived with this test and sum up these ratios for all components of the mixture.

### Grouping of unknown mixtures of unknown substances

Grouping pesticides by effects on indicator systems is of high importance because to date combination toxicology is facing a generic problem: for many potentially toxic substances produced or just present at relevant amounts the mechanism of action is unknown and their toxicity has not been evaluated. According to data from the US EPA, for about 80% of substances produced at significant amounts no toxicity information is available. For pesticides this percentage goes down to about 40%. For pesticides the knowledge level is relatively good but far from being sufficient. The U.S. EPA has launched a set of projects on chemicals risk assessment with the aim of developing methods for the prediction of chemical toxicology suitable for dealing with a high number of substances, to improve incorporation of molecular toxicology and computational science, to reduce standard rodent toxicology tests and to increase cost efficiency [39]. Complex data on chemical structures, results of high throughput screening and rodent test data are evaluated in order to identify groups of chemicals characterised by their effects on cellular pathways and more generic health endpoints.

With respect to mixtures the approach is based on the identification of relationships between the structure of a substance and its toxicity. If structural features of a substance group are correlated with toxicity revealed from *in vitro* assays determined using high throughput screening, and the same structural features are correlated with *in vivo* toxicity test results, *in vitro* assay results or even structural features might be sufficient to predict the mechanism of action and health effects. The approach aims at predicting the mode of action and/or toxicity of a high number of chemicals within a short time period. In the context of mixtures of chemicals with unknown mode of action the methods might be suitable to sort the compounds of a mixture by predicted modes of action in order to define groups of chemicals for which additive combination toxicology approaches, such as concentration or dose addition, or hazard index related methods, can be applied.

## Discussion

In tackling the health risk associated with combined exposure to mixtures of chemicals in the environment, the food chain and consumer goods, regulatory bodies will have to deal with several problems:

• Setting maximum acceptable threshold concentrations or acceptable daily intakes (ADIs) for certain chemical groups with a common mechanism of action such as organophosphates would be a first step forward, but would disregard most of the mixture constituents as the majority of active substances in a sample usually belong to different chemical groups (an example is given in Table 1).

**Table 1:** Plant protection products found in German fruit with corresponding health effects

active substance	chemical group*	pesticide type*	target	health issues*					
				carc	end	repr	AChE	neuro	resp
Endosulfan-sulfate	organochlorine	acaricide, insecticide	neurotoxic, affects the transfer of nerve impulses in insects and mammals	?	?	-	no	yes	-
Chlorpyrifos-ethyl	organophosphate	insecticide	AChE inhibitor, causes dysfunction of the nerval system	no	?	yes	yes	no	no
Chlorpyrifos-methyl	organophosphate	acaricide, insecticide	AChE inhibitor, causes dysfunction of the nerval system	no	no	-	yes	?	no
Triadimefon	triazole	fungicide	disrupts membrane function	?	?	yes	no	?	-
Trifloxystrobin	strobilurin	fungicide	inhibits electron transfer and respiration	no	-	yes	no	no	-
Boscalid	carboxamide	fungicide	inhibits sperm germination	?	no	?	no	no	-
Cyprodinil	anilinopyrimidine	fungicide	blocks certain synthesis pathways within the cells	no	-	?	no	no	yes
Dimethomorph	morpholine	fungicide	lipid synthesis inhibitor	no	-	?	no	no	no
Fenhexamid	hydroxyanilide	fungicide	Disrupts membrane function, inhibits spore germination	no	-	no	no	no	no
Fludioxonil	phenylpyrrole	fungicide	inhibits phosphorylation of glucose	?	-	?	no	no	no
Fluopicolide	benzamide	fungicide	protectant	?	-	no	no	no	no
Metalaxyl	phenylamide	fungicide	protectant suppressing infections, sporangial formation and mycelial growth	no	-	no	no	no	-
Thiametoxam	neonicotinoid	insecticide	affect the central nervous system by binding to an postsynaptic ACh receptor	?	no	no	no	no	?

• Additive approaches based on grouping substances by effects (e.g. summing up doses of compounds causing dysfunction of the nervous system in humans but acting via differing mechanisms) requires testing results obtained under comparable conditions and evaluating effects on a common human health endpoint. Such data are not available in most cases, and, again, only a part of the mixture constituents would be considered (Table 1).

• Using the hazard index approach for assessing the risk to human health is practicable and transparent but the question arises whether HQs of all mixture compounds should be added, or, alternatively, HQs of substances grouped on the basis of their relevance for a certain health effect. Krautter et al [40]

• describe an evaluation scheme for pesticide mixtures considering both threshold exceedances (ARfD, ADI, MRLs) and HIs, the latter calculated as the sum of HQs of all substances in the sample (See http://www.greenpeace.de/fileadmin/gpd/user_upload/themen/umweltgifte/greenpeace_bewertung_pestizide_neu.pdf)

• Adding HQs of all compounds found has the advantage that it is transparent and easy, but has the disadvantage that improving the detection limit of the analysis and thus identifying a high number of compounds present at very small amounts might increase the overall hazard index, whereas an analysis of the same sample with higher detection limit would indicate a lower risk. Thus, the overall estimated risk of the mixture is only dependent on the detection limit and not on the actual biochemical mechanisms that determine toxic potency.

• Adding HQs of compounds within a common health effect would probably reveal additional information on specific risk of the mixture, but has the disadvantage that for most active substances currently in use the dataset on health effects is incomplete: for three of the 13 compounds in Table 1 it is known that they have no endocrine disrupting effects, for three it is known that they might have these effects, but it is not clear, and for seven others no data on endocrine disruption are available.

• A further problem connected with the use of the hazard index approach for the risk classification of mixtures is that a suitable reference has to be chosen. It has to be decided whether the HQs should be calculated as a residue versus MRL or a predicted daily dose versus the ADI or both, and, in the case of dose versus ADI, whether the doses and ADIs for adults or children should be applied.

• The lack of knowledge about actual or potential synergistic interactions is a general problem. Legislation can deal with this problem by diminishing reference concentrations or doses of no concern by a factor assumed to be protective, but whether this factor should be 2, 3 or 10 is a matter of speculation.

• Existence of toxic metabolites and potential biochemical interaction among the mixture components influencing the level of metabolic activity complicates further the risk assessment of mixtures. This complication is further increased when the biological half-life of the mixture components varies significantly. Understanding the nature of component interactions and the related biokinetics is a key requirement for assessing effectively chemical mixtures with these characteristics.

To date, the effects of chemical mixtures are dealt with by the regulatory framework in the USA, but not in the European Union. The International Programme on Chemical Safety of the World Health Organization (WHO IPCS) has developed guidelines with a view to enlarging their applicability to its domain of competence (worldwide); both of these initiatives, however, are limited to human health effects. The current thinking within the European Commission and in the relevant scientific and industrial community in Europe is that any European initiative towards the establishment of guidelines regarding the assessment of chemical mixtures should address equally the health of both humans and the environment [41]. Such guidelines could be built around a core of tools, methods and approaches common to mammalian and ecological toxicology, the most important of which are described in this paper.

What type of general principles can be included in a European Commission recommendation in order to address combination effects?

Clearly, currently there is a need to strengthen the legal mandate for risk assessment of mixtures. In this context, mixture effects need to be taken into account holistically, drawing useful lessons from the CIRCLA legislation in the United States, targeting Superfund sites. If an overarching framework is set based on general guidelines and underpinned by as much scientific rigor as possible, then more sectorial implementation could vary to adapt to the twenty one different pieces of European Community legislation pertinent to the issue of chemical mixture safety, including legislation on chemicals (REACH), consumer articles (Consumer Strategy), plant protection products (Plant Protection Products Directive), biocides (Biocides Directive), water quality (Water Framework Directive and Marine Strategy), and air pollution (Air Quality Framework Directive). The forthcoming WHO guidelines for indoor air quality, currently under development in conjunction with the European Commission, would be an opportunity to incorporate thinking about risk assessment of mixtures in a sectorial piece of legislation affecting almost all the European population for more than 80% of the time. In this context, a Europe-wide data center on human exposure to environmental chemicals could provide the necessary scientific support for effective risk assessment of chemical mixtures. For this purpose, the European Commission’s Joint Research Centre in collaboration with the European Environment Agency has been working towards the development of this data center since 2008. So far, the basic concept for the data center has been developed. The available monitoring data for cumulative exposure assessment are expected to be pulled together by the EEA topical centres (on air, water, soil) in the course of 2011. In the food products domain, the European Food Safety Authority (EFSA) has recently initiated similar efforts, while the European Chemicals Agency (ECHA) is in the process of populating the IUCLID 5 database with physical/chemical and toxicity data on industrial chemicals currently in the European market. Setting up an interagency group bringing together the efforts of all the European Union organs would be the most efficient way towards coordinated action to assess cumulative exposure assessment to chemical mixtures.

A possible way forward to overcome the current gaps in knowledge that could act as obstacles to the definition of a plausible regulatory approach to chemical mixture risk assessment would be to use a tiered approach as follows:

(a) Use dose addition to calculate a hazard index taking into account interactions (eq. 16) as default option for hazard quantification and risk assessment. This approach could be employed as default first-tier approach for mixtures risk assessment. The formulation of the hazard index given in eq. 16 allows taking into account the non-linear effects from the interaction of mixture components if the necessary information is available, while simplifying down to a simple dose addition if no interaction data exist. Such an approach would be in line with the current practice across the Atlantic, as the interaction-based hazard index has been developed and used extensively by the US Environmental Protection Agency. Overall, it would give a reasonable approximation to the toxic potency of a mixture if the necessary data are available; if not, it would still allow conservative assumptions about effects of combined exposure to multiple chemicals and interactions among the mixture components.

In data-rich situations use more sophisticated tools, including mechanistic, biology-based modeling that takes into account the biologically effective dose of the mixture components at the target tissues and incorporates system-wide response data across the dose-response range using information derived from –omics technologies – the connectivity approach [42]. This more complex yet plausible methodology would be in line with the United States Academy of Science 2007 report towards toxicity testing in the 21^st^ century [43].

The tiered approach outlined above ensures that it can be readily applied in the current regulatory context and yet opens up the way to incorporating scientific state of the art in the near future. A key development that could be implemented readily in already existing pieces of legislation (during their regular review process) would be to demand that the collected toxicity data be amenable for mixture toxicity assessment. This means essentially three things:

i. that toxicity data are recorded and documented in a coherent uniform way, independent of specific regulatory context;

ii. that emphasis should be given to the full range of the dose-response function, and in particular below the NOAEL or DNEL (derived no effect level, as used in REACH); and,

iii. that benchmark approaches should be given preference over NOAELs or NOECs in setting up regulatory safety limits for chemical substances.

In addition to immediate regulatory change that could facilitate a rational assessment of chemical mixture toxicity as described above, further research is needed targeting at least the following key issues:

• development of dedicated exposure assessment strategies for environmental chemical mixtures (e.g. following the example of the US Geographic Survey collection of water samples analyzing a large number of chemicals)

• analysis of the determinants of synergistic action (i.e. giving answers to the question ‘when is it likely that a mixture acts synergistically?’)

• identification and assessment of the chemicals that contribute most to cumulative risk on the basis of integrated assessment under specific exposure scenarios of relevance to sectorial policies

## Conclusions

This paper gives a comprehensive overview of the currently available methodological and computational tools for assessing the health effects of chemical mixtures with a view towards applying them in chemical safety regulation. The salient pitfalls in converting scientific knowledge on combination effects of co-exposure to multiple chemicals are discussed and a tiered approach to mixture risk assessment is outlined for use by regulators and policy makers.

An EU strategy for assessing and managing combination effects of chemicals in the environment could comprise the following elements:

– development of guidelines on general principles; there is a need to explain why the combination effects of chemicals have to be addressed effectively from concerted policy and regulatory action based on robust scientific grounds; there is a need to bring out the problems based on scientific evidence and to start assessing these combination effects.

– use of the methodological tools available as outlined in this paper. For data poor situations, dose addition could be used as default option in a tiered approach that moves from dose addition to fully mechanistic analysis of the possible interactions among the components in a chemical mixture [43].

– resolution of the difficulties with dealing with the different pieces of legislation related to the health effects of environmental chemical mixtures and achievement of a holistic approach. There is a clear need to address the issue in a coordinated fashion among the European Commission services responsible for legislation across different media with a view to identifying the practical steps towards achieving this integrated approach.

– further scientific research aiming at getting more precise information on cumulative exposure to chemical mixtures in the environment and using better the information collected through different pieces of legislation in order to inform policy makers’ choices and enable them to prioritize.

## Competing interests

The authors declare that they have no competing interests.

## Authors' contributions

DAS supervised the study that led to this manuscript and wrote explicitly the introduction, biological modeling, discussion and conclusions sections; he also takes the responsibility for drafting the overall manuscript as is. UH provided the material for the sections reviewing the current methodological approaches for cumulative risk assessment of chemical mixtures and contributed to the discussion.
